# Shared Decision-Making in Oncology – A Qualitative Analysis of Healthcare Providers’ Views on Current Practice

**DOI:** 10.1371/journal.pone.0149789

**Published:** 2016-03-11

**Authors:** Wiebke Frerichs, Pola Hahlweg, Evamaria Müller, Christine Adis, Isabelle Scholl

**Affiliations:** 1 Department of Medical Psychology, University Medical Center Hamburg-Eppendorf, Hamburg, Germany; 2 Department Health Sciences, Hamburg University of Applied Sciences, Hamburg, Germany; US Army Engineer Research and Development Center, UNITED STATES

## Abstract

**Background:**

Despite an increased awareness of shared decision-making (SDM) and its prominent position on the health policy agenda, its implementation in routine care remains a challenge in Germany. In order to overcome this challenge, it is important to understand healthcare providers’ views regarding SDM and to take their perspectives and opinions into account in the development of an implementation program. The present study aimed at exploring a) the attitudes of different healthcare providers regarding SDM in oncology and b) their experiences with treatment decisions in daily practice.

**Material and Methods:**

A qualitative study was conducted using focus groups and individual interviews with different healthcare providers at the University Cancer Center Hamburg, Germany. Focus groups and interviews were audio-recorded, transcribed and analyzed using conventional content analysis and descriptive statistics.

**Results:**

N = 4 focus groups with a total of N = 25 participants and N = 17 individual interviews were conducted. Attitudes regarding SDM varied greatly between the different participants, especially concerning the definition of SDM, the attitude towards the degree of patient involvement in decision-making and assumptions about when SDM should take place. Experiences on how treatment decisions are currently made varied. Negative experiences included time and structural constraints, and a lack of (multidisciplinary) communication. Positive experiences comprised informed patients, involvement of relatives and a good physician-patient relationship.

**Conclusion:**

The results show that German healthcare providers in oncology have a range of attitudes that currently function as barriers towards the implementation of SDM. Also, their experiences on how decision-making is currently done reveal difficulties in actively involving patients in decision-making processes. It will be crucial to take these attitudes and experiences seriously and to subsequently disentangle existing misconceptions in future implementation programs.

## 1. Introduction

Patient-centered care and patient involvement in healthcare decisions have become key components in high-quality modern healthcare [1–6]. As the traditional physician-patient relationship has changed, active participation and engagement of patients in medical decisions are important steps towards quality healthcare [7, 8].

Shared decision-making (SDM) has been fostered as the pinnacle of patient-centered care [9]. SDM is defined as an interactional process in which the patient and the clinician aim to reach a decision together that is based on shared information and the best available evidence [10]. In the course of this process, the clinician supports the patient in weighing the risks and benefits of different diagnostic or treatment options, in order to come to a shared and informed decision [11]. SDMthus encourages the interaction between the clinician and patient by exchanging information and individual preferences [12]. SDM has been associated with the following: decreased fear and depression [13]; improved quality of life [14]; increased patient and treatment satisfaction [1]; reduction of overuse of treatment options and unwarranted practice variation [15–17]; and contribution to job-related satisfaction among clinicians [18, 19].

SDM has been reported as particularly valuable in cases of medical uncertainty considering disease and treatment outcomes, e.g. in cancer care [15, 16]. Several studies show that cancer patients have a preference for involvement in decision-making [7]. However, discrepancies between preferred and actual perceived level of involvement still exist [20–22]. Therefore, the engagement of patients and their support in the decision-making process are essential parts of cancer care [23].

Despite a significant body of evidence on SDM [24] and endeavors on the health policy level to foster SDM in Germany (e.g., several large funding programs focusing on SDM and patient-centered care [2]), its implementation into clinical practice remains a challenge [17, 25, 26]. Doubts about the benefits of SDM continue to exist among clinicians (e.g. that patients do not want to be involved [26], or that there is a lack of applicability due to time constraints [12, 18], patients’ characteristics [27], or cancer characteristics [1]). Furthermore, research has demonstrated that patients’ preference for SDM and their needs and wishes for information and participation are often underestimated by physicians [18, 28, 29]. In light of the potential benefits of SDM and the patients’ wishes for active participation in treatment decision-making, the promotion of SDM in daily healthcare practice seems to be a feasible step.

However, in order to engage patients in the decision-making process, healthcare providers (HCPs) need to understand the concept of SDM and how to apply it in routine care [1]. While it has often been highlighted that it is important to engage HCPs other than physicians in SDM [1, 30], most research on HCPs’ attitudes on and experiences with SDM has focused on physicians only, both in Germany [28, 31] and internationally [1, 26, 29, 30, 32]. Thus, a research gap exists regarding the views of other HCPs (e.g. nurses, psycho-oncologists). Furthermore, research on current perceptions on SDM in oncology in Germany is missing. However, to successfully implement SDM in routine cancer care in Germany, it is indispensable to understand the whole process of decision-making from a multidisciplinary perspective [26, 33, 34]. Only if implementation strategies are grounded on the attitudes and experiences of HCPs in a specific setting and health care system, they are likely to be effective [35].

Thus, the aim of this study is to explore the views of different HCPs regarding the current practice of decision-making in oncology in Germany. This includes the following research questions:

- What are the attitudes of the different HCPs towards SDM?
- What are HCPs’ experiences with treatment decisions in current practice?

## 2. Materials and Methods

### 2.1. Study Design

A qualitative study was conducted analyzing data from semi-structured focus groups and interviews with different HCPs using conventional content analysis [36]. Qualitative research is an ideal approach for exploring new areas of study [37] and to uncover beliefs, values, and motivations [38].

### 2.2. Setting and Subjects

The study was carried out at the University Cancer Center Hamburg (UCCH), a substructure of the University Medical Center Hamburg-Eppendorf (UKE), Germany. The UCCH is a comprehensive care and research center including all medical departments of the UKE that are involved in diagnosis and treatment of cancer. Thus, the sample consisted of HCPs working at the UCCH.

### 2.3. Focus Groups

We planned to conduct four focus groups (n = 4) of 90 to 120 minutes with 8–10 participants per group including HCPs with different clinical backgrounds. One group was planned with assistant physicians (also known as junior doctors or resident physicians in the UK and US respectively), one with senior physicians (also known as consultant or chief resident/senior staff member in the UK and US respectively), one with nurses, and one with other HCPs (e.g., psycho-oncologists, physical therapists). No further inclusion criteria were specified. To allow HPCs in more junior positions to express their thoughts sincerely and openly, participants were assigned to focus groups according to their professional background and hierarchical position (e.g. assistant physicians and senior physicians were assigned to separate focus groups).

Focus group discussions have been shown to be a useful approach for actively exchanging ideas and opinions among participants, for exploring participants’ knowledge and experiences, and to assess attitudes and needs of staff [39].

### 2.4. Individual Interviews

As it would have been difficult to schedule a focus group for head physicians (comparable to senior or principal consultants in the UK and US respectively) and management staff (e.g. nursing management or quality management), individual face-to-face interviews were conducted. Also, it was expected that participants would not be as open and honest within a focus group discussion, if their supervisor would participate as well. No further inclusion criteria were specified, except that participants were either a head physician of one of the UCCH departments or were in a management position associated with the UCCH. Individual interviews were sought to last approximately 45 minutes.

### 2.5. Recruitment

Participants for this study were recruited in collaboration with cooperation partners at the UCCH.

### 2.6. Focus Groups

Relevant participants were identified through several steps and contacts: focus groups for assistant and senior physicians were recruited 1) through the head physicians of relevant UCCH departments, who forwarded the invitation to their staff, and 2) by directly contacting physicians of the UCCH, who were familiar to the authors due to participation in previous project phases. Nurses were recruited through nursing management staff, which was contacted through the UKE website and again, invited by email. Further HCPs were identified through the UKE website and then invited by email directly.

### 2.7. Individual Interviews

Recruitment of participants took place by identification of relevant head physicians and management staff through the UKE website followed by a direct invitation by email. In case of no answer, a reminder was sent twice. If a reply remained to be absent after the second reminder, participants were marked as not interested.

### 2.8. Data Collection

Focus groups took place between April and June 2014, interviews between May and July 2014.

Before the conduction of the focus groups and interviews, participants received an information sheet and signed an informed consent for participation. Additionally, all participants were asked to complete a questionnaire on demographic and occupational data. They were offered a compensation of twenty-five euros for their participation in the study.

Two researchers (IS and PH) led the focus group discussions, which were audio-recorded. An additional researcher (WF) took minutes to clarify eventual difficulties during the transcription of the audio-recordings. First, participants were asked to give their opinions about how decision-making should take place. To prompt this discussion, participants were shown two humorous cartoons, one showing paternalistic decision-making and one showing informed decision-making. Participants were asked to have a look at the cartoons and to express their opinion on the cartoons. Then, results from a previously conducted state analysis at the UCCH were presented and discussed. Next, a discussion on possible needs and interventions to facilitate SDM followed.

One moderator (IS or PH) conducted the individual interviews. First, interviewees were asked to share their perception on how decisions are made within their department. This was done by either presenting the results of the previous actual state analysis or by comparing daily practice with a prototype model of SDM [40]. Second, barriers to SDM and possible interventions to facilitate SDM were discussed.

For this paper, only responses related to our aims exploring the attitudes and experiences of different HCPs towards SDM were analyzed.

### 2.9. Data Analysis

Focus groups and individual interviews were audio-recorded. In a first step, these audio-recordings were transcribed and anonymized to rule out any possibility of identification of the participants or other people and settings (e.g. clinics, specific HCPs). Transcription was based on guidelines for simple transcription, i.e. the transcripts prioritized content and readability, e.g. omission of most para-verbal and non-verbal elements of communication, approximation of colloquial language to standard language [41]. Transcription was supported by the software f4 (dr. dresing & pehl GmbH, Marburg, Germany). Anonymized transcripts were not returned to participants for corrections. Anonymized transcripts were imported into MAXQDA software (version 10; VERBI GmbH, Berlin, Germany). The analysis followed the principles of qualitative content analysis described by Hsieh & Shannon using a conventional approach [36] and was carried out by two researchers. It consisted of the following steps. First, the complete data set was read to gain an overview of the data (WF) [42]. Second, operational definitions were developed for the terms “experiences” and “attitudes” (WF). Third, 50% of the data (i.e. 2 focus groups and 8 interviews) were read again and initial inductive categories were developed using a paragraph-by-paragraph approach (WF). Within this approach, text that could not be categorized into the previously developed categories was coded with a new category. Fourth, categories were revised and systematized by developing subcategories and clustering categories (WF). Fifth, the preliminary scheme developed in steps one to four was discussed with a second researcher (IS) and subsequently the scheme was revised. Sixth, the categorization of the data was revised with the adjusted scheme (WF). Seventh, the data interpretation was cross-checked by a second researcher (IS), which allowed valuable discussions followed by shared adjustments of the category scheme (IS, WF) [43]. Eighthly, the remaining 50% of the data were coded and additional categories were integrated where necessary (WF). As a last step, the final category scheme was discussed and revised (IS, WF).

Memos were written during the coding process to capture impressions and to facilitate the identification of themes and patterns. Additionally to the qualitative analysis, descriptive statistics were calculated.

### 2.10. Ethical Approval

The study was carried out in accordance with the Code of Ethics of the Declaration of Helsinki and was approved by the Ethics Committee of the Medical Association Hamburg, Germany. Participants provided written informed consent to participate in this study. The Ethics Committee approved this consent procedure.

## 3. Results

### 3.1. Characteristics and Description of Focus Groups and Interviews

Overall, N = 43 HCPs participated in the study. *Four* focus groups were conducted with a total of *N = 25* participants: a) assistant physicians (N = 8), b) senior physicians (N = 5), c) nurses (N = 4), d) other healthcare professionals (N = 8, including 2 nurses). Apart from a range of different healthcare professionals (e.g. psycho-oncologists, physical therapists), the last group also included two nurses, who were not available at the date of the focus group for nurses. The mean duration of the focus groups was 115 minutes and the mean amount of participants was 6. 16 (64%) of the focus group participants were female. Additionally, *17* interviews with a total of *N = 18* head physicians (one interview was done with two head physicians) and other HCPs in management positions were carried out. The mean duration of the interviews was 48 minutes. 4 (22%) of the interview participants were female. Interview participants were older and had more working experience in cancer care than focus group participants. Table 1 shows an overview of participants’ characteristics.

**Table 1:** Descriptive characteristics of the total sample (N = 43), the focus group sample (N = 25) and the interview sample (N = 18).

	Focus groups (N = 25)	Interviews (N = 18*)	Total sample (N = 43)
Age (mean (SD) in years)	40.68 (11.45)	52.94 (6.17)	45.81 (11.31)
Working experience in cancer care (mean (SD) in years)	13.60 (10.47)	23.47 (10.59)	17.45 (11.47)
Duration of employment at UKE (mean (SD) in years)	9.55 (8.69)	15.50 (7.55)	12.04 (8.66)
Profession (N (%))			
Physicians	13 (52.0)	14 (82.4)	27 (64.3)
Nurses	6 (24.0)	2 (11.8)	8 (19.0)
Psycho-oncologists	3 (12.0)	0 (0)	3 (7.1)
Other professions	3 (12.0)	1 (5.9)	4 (9.6)

Legend:

*Sample size varies between 18 and 17 due to missing values.

SD = standard deviation.

Generally, we observed that topics discussed in the interviews varied greatly. Some interviewees’ responses focused primarily on multidisciplinary team meetings (MDTMs), or on barriers and facilitators. Both aspects have not been the focus of this research. In order to ensure anonymity, we did not match descriptive characteristics of participants to their contributions in the focus groups and interviews.

### 3.2. Qualitative Analysis

In the analysis of the 4 focus groups and 17 interviews, two main themes were identified: attitudes towards SDM and experiences with decision-making.

Regarding the attitudes towards SDM, three categories have been extracted:

- What is SDM?
- Who should decide?
- In which situation should SDM take place?

Regarding experiences with decision-making, two categories have been generated to explore how decision-making currently takes place.

- Experiences with decision-making on the micro level
- Experiences with decision-making on the meso level

The micro level refers to decision-making for the individual patient or in situations with a HCP, whereas the meso level describes decision-making concerning clinical characteristics and structures, e.g., healthcare institutions or integration of multidisciplinary team meetings [2, 44].

### 3.3. Attitudes

#### What is SDM?

Participants had different opinions about what SDM really is. In most cases, participants reported that patients should be involved in decision-making and that SDM represents a process. Still, the extent to which a patient should be involved varied greatly between mere presence of the patient (e.g., *“The medical round is actually the ideal forum to realize SDM*. *Nurse*, *patient and possibly a relative on a chair next to them*, *and the physician*. *They are standing there and talk about the patient*: *‘What type of findings did we get*? *What meaning do they have*?*’ And so on*.*”*) and real involvement (e.g., *“[SDM is] a setting at eye-level*, *with the physician*, *the patient and relatives*, *where the consultation takes place neutrally*, *about options and treatment alternatives*, *what they mean and their risks and benefits*, *and that the patient is included with his or her entire being*.*”*).

Some understood SDM and patient involvement as a form of informed consent (e.g., “Every patient needs to be sufficiently informed to sign the informed consent form. And by doing so he is involved”).

#### Who should decide?

The attitudes towards decision-making authority also strongly varied among the participants. When dividing decision-making into the three most prominent models (the paternalistic model, the shared model and the informed model), most answers could be assigned towards the paternalistic model of decision-making. Particularly dominant was the attitude that physicians are able to influence the decision of a patient (e.g., *“I believe [45] WE lead the patient, we decide, what he is going to do. And it depends on how we sell this to him.”*). Physicians reported that they could not be neutral throughout the deliberation process (e.g., *“I don’t think it is possible to inform a patient in a way that you do NOT influence him with your own thoughts*.*”*). Some participants reported that decision-making should be an informed-choice process (e.g., *„The patient is the one who is going to decide and we only support him*. *That’s how I understand it*.*“*).

#### In which situation should SDM take place?

Participants had various ideas on when SDM should take place. These depended largely on the characteristics of the patient (e.g., cognitive abilities; actively asking for involvement) or when the patient is well informed in order to decide (e.g., *“[It is important] that the patient comes with a certain level of information*. *Only then the patient can decide*.*”*). Also, time was rated as an important contributor to SDM (e.g., *“If time is plentiful*, *then you should do it [SDM]*. *But if you don’t have time*, *then you as a physician (…) need to take the best decision for the patient*.*”*). Furthermore, many participants reported that SDM depends on whether several treatment options are available and that the possibility of applying SDM depends on the type of cancer or disease (e.g., one HCP said: *“In our department it’s about life or death*. *SDM can be done by someone else (…)*, *maybe someone who is in cosmetic surgery*.*“*, and another HCP added: *“Or prostate cancer for example*, *this has time*.*”*).

### 3.4. Experiences

#### Experiences with decision-making on the micro level

Providers stated that patients are sometimes not satisfied with the information received or with the decision taken (e.g., “And three days after the surgery […] the first sentence they [patients] write on a piece of paper as they cannot talk or eat: ‘I should have never done this!’, and for ME, it is obvious that there has not been done a good job previously.”). Many providers reported insufficient information exchange. This was particularly the case for consultations about possible risks and impairments (e.g., decreased quality of life) or about treatment options (e.g., “But this complex therapy and that often something can go wrong, they [patients]are not told about.” or “The outcome of chemotherapy […], that is, unfortunately, often missing. Because numbers are not available or […] because one is afraid to tell the patient.”). Also, many participants experienced that the information delivered to the patients during consultations was too much or too complex to understand (e.g., “It is certainly the case, that for many patients, the information is overwhelming.”). In contrast, some participants experienced patients who joined consultations already well informed (e.g., sought available information from the internet or from prior consultations in other clinics before having consulted the UCCH).

Experiences regarding the discussion of treatment options also varied greatly. Overall, the majority of participants reported that mostly only one treatment option was discussed with the patients. Reasons were that either no medically “good” alternative was available or that too many alternatives existed. In cases in which different treatment options were discussed, the patients have been actively asking for an alternative (e.g., *„The patient asks at the first mentioning of a suspected [cancer] diagnosis*, *‘What does this mean for me*? *Which treatment alternatives are there*?*’ […]*. *And then the physician will explain them [the options] to him*.*“*).

When analyzing the category physician-patient relationship, often reported experiences included poor physician communication styles, the physicians being able to influence the patient during the process of decision-making, and patients reacting with fear or being overwhelmed during consultations and not being able to understand the forwarded information. Still, other participants also reported positively about the interaction with the patient, stating a very open and honest communication and a trustworthy relationship, which was developed over time. However, the physician-patient relationship, the patient’s behavior, and whether SDM took place or not, strongly depended on individual patient characteristics and their needs (e.g., cognitive abilities; expressing a wish for a certain therapy). Although some participants reported decisions to be taken within a shared or informed approach, most reported the decision-making to be paternalistic. HCPs explained that this was due to their impression that patients did not want to participate in the decision-making process.

Considering the involvement of others, HCPs stated that relatives are often included, whereas nursing staff or other HCPs are less likely involved in the decision-making process.

#### Experiences with decision making on the meso level

On the meso level, participants experienced structural influences on decision-making processes. Many participants reported the multidisciplinary communication between clinics and HCPs to be poor, and economic and hierarchy constraints to be influential (e.g., *“Economic pressure [to take certain decisions]*. *Very clearly*, *economic pressure*.*”*). Time constraints were the most often reported experiences among all HCPs. One of those experiences was related to time pressure regarding certain cancer types that require faster decisions than others. The other experience was that doing SDM takes more time. However, many participants did positively report about time factors. They deliberately take time for their patients, do not put them under pressure during decision-making, or stated a better outcome when taking their time (e.g., one HCP said: *“I would also SWEAR when only taking ONCE the time*, *then […]“*. Other HCP added: *„Then things just run*.*“*).

Regarding experiences with multidisciplinary team meetings (MDTMs), most physicians reported that “decisions are taken” rather than “recommendations given”. Furthermore, few participants experienced that patients’ individual preferences were discussed in MDTMs.

## 4. Discussion

The present qualitative study explored the attitudes and experiences of 43 HCPs towards SDM in oncology by conducting focus groups and individual interviews. The findings showed that participants’ attitudes towards SDM varied greatly. This variation is particularly present when discussing the definition of SDM, the attitude towards the degree of patient involvement in decision-making and assumptions about the situations in which SDM should take place. Overall, participants had a generally positive attitude towards SDM in oncology. However, many participants also reported that SDM might not always be the appropriate method in the process of decision-making. This was also reported to depend on the individual patient’s needs and tumor characteristics. Our findings are new for Germany and at the same time in line with previous studies in other countries, reporting similar challenges towards the implementation of SDM [27, 46].

Participants only rarely described SDM in the way it is defined in the international literature on SDM (cf. Introduction [10–12]). A few participants expressed that if a patient agrees to the presented treatment, e.g., by signing an informed consent form, he or she is involved and a shared decision is made. This finding suggests that there is still a lack of knowledge among some HCPs regarding SDM, its steps and best practice. Such misconceptions might hinder a successful implementation of SDM in routine practice [24, 47]. Even though most participants deemed patient involvement important, no clear consensus on the degree of involvement existed. Other research stated the difficulty to really define the degree of involvement, whether from the perspective of the provider or from the patient [48].

The concept of SDM is fundamental to patient-centered care [24]. However, many physicians reported that they are able to influence a patient in his or her decision. This is in line with the results by Karnieli-Miller & Eisikovits [49], which showed that a variety of persuasive strategies were used by physicians in treatment decision-making. This suggests that one needs to be especially careful when assessing patients’ views on whether a decision was taken together or by the HCP alone, or when assessing the perceived degree of involvement in the decision-making process. Patients were found to have difficulties to realistically identify the decision-maker and to tell whether clinicians communicate objectively or in a paternalistic way. Both approaches can be falsely judged as a SDM process by the patient [50–52]. Previous research investigated that the “physician’s attitude towards SDM” was the most significant variable to stimulate the patients’ intention to engage in SDM [53]. Consequently, one has to take the provider’s individual capability to influence the patients’ behavior into account when implementing SDM trainings for providers. SDM should not be misused to influence a patient’s decision towards the provider’s own preferences.

The provision of patient information was experienced differently by study participants. Many reported experiences included cases in which information was either perceived as insufficient or as an overload. In consequence, the patients reacted overwhelmed or with fear. Other studies suggest tailoring the information based on the clinical situation and the individual patient. Some patients might only want basic information when faced with the diagnosis of cancer, whereas others wish for more in-depth information and support during the deliberation process [1, 46]. Furthermore, quantitative decision support systems have been described as useful tools that can be helpful for clinicians to offer patients sound information in terms that patients can understand, while engaging them in the decision-making process [34]. In our study, participants reported that SDM does not take place due to patients not wanting or not being able to decide. Providers expressed that alternative options were only rarely discussed. Previous research has found that the majority of patients is still motivated to discuss options and receive information from their physicians, even though they may not wish to make the final decision [7, 54, 55]. Studies have also shown that providers tend to underestimate the degree to which patients want to be involved in decision-making [56, 57]. Thus, it is important that HCPs inform their patients about the different available treatment options and their consequences, and also assess each patient’s preference for involvement in decision-making. If, after these steps, a patient still wants to delegate the final decision to his or her HCP, then the process of the decision is still shared, and a respectful relationship maintained.

This study has several limitations. First, the sample was a convenience sample recruited through several pathways. As recruitment also took place through forwarded emails, the research team was not completely in control about who and how many HCPs were invited by email. Also, it could have been the case that head physicians, for instance, who may have forwarded the email specifically to physicians, who are more open to the concept of SDM, might have biased the study sample. Further, it can be the case that physicians were explicitly required to participate by their supervisor. This was especially obvious in the focus group of assistant physicians as two reported that their line manager had asked them to participate. Although this recruiting process might induce such biases, it, more importantly, enabled the research team to reach a greater diversity of participants. Second, as in two focus groups the number of participants was lower than eight, insufficient recruitment of subjects could be a potential bias. However, as participants cancelled shortly before conduction of the focus groups, or did not cancel at all due to clinical emergencies or on call-duties, conducted recruitment is thought to be sufficient for a medical setting. As focus groups with fewer participants still achieved a variety of results that did not differ from those of other focus groups, a bias of over- or under-reporting is unlikely.

A main strength of the present study is the inclusion of different HCPs from a variety of clinical backgrounds, therefore reflecting a multidisciplinary view on decision-making. Previous research suggested to take the views of different HCPs on SDM into account as prior focus was mainly on physicians’ perceptions. Including different HCPs is a step towards assessing and understanding the whole decision-making process, not only it's barriers, as proposed by researchers [26, 27, 33]. At the same time, it must be noted that due to confidentiality, this study did not allow matching the characteristics of participants (e.g. age, profession, role) with their contributions to the focus groups and interviews. This should be taken into account in future studies, in order to allow a more detailed analysis of potential differences among HCPs with different characteristics. Also, the role of professional development and training in medicine, nursing and other healthcare professions should be further explored in a subsequent study. The methodological approach including data analysis conducted by two researchers, and multiple revisions and crosschecking can be seen as a further major strength of this study. Finally, this study investigated a topic that has received only limited attention so far. It therefore adds new knowledge that can be used for the implementation of SDM in oncology in Germany and possibly in other countries with similar healthcare systems.

## 5. Conclusion

Despite SDM being a possible “gold standard” for a high quality cancer care, German HCPs gave mixed reports about their current attitudes and experiences with SDM in daily practice. SDM largely depends on the clinicians’ attitude towards SDM, patient and disease characteristics and structural constraints, such as time or poor communication between cancer care clinics. Above mentioned factors currently function as barriers towards SDM implementation.

In order to develop a successful implementation program, it will be crucial to take these attitudes and experiences into account and to subsequently disentangle existing misconceptions, e.g., through SDM trainings for providers. Finally, the results indicate that it could be fruitful to empower patients to actively ask questions and express their preferences for their preferred role of participation, as some providers seem to misinterpret these preferences.
